# Editorial: Inference, Causality and Control in Networks of Dynamical Systems: Data Science and Modeling Perspectives to Network Physiology With Implications for Artificial Intelligence

**DOI:** 10.3389/fphys.2022.917001

**Published:** 2022-05-11

**Authors:** Paul Bogdan, Plamen Ch. Ivanov, Sergio Pequito

**Affiliations:** ^1^ Ming-Hsieh Department of Electrical and Computer Engineering, University of Southern California, Los Angeles, CA, United States; ^2^ Keck Laboratory for Network Physiology, Department of Physics, Boston University, Boston, MA, United States; ^3^ Harvard Medical School and Division of Sleep Medicine, Brigham and Women’s Hospital, Boston, MA, United States; ^4^ Institute of Solid State Physics, Bulgarian Academy of Sciences, Sofia, Bulgaria; ^5^ Delft Center for Systems and Control, Delft University of Technology, Delft, Netherlands

**Keywords:** fractional-order dynamic models, artificial Inteligence-AI, physiology, multifractal networks, neuro-inspired artificial intelligence

A fundamental problem crisscrossing the fields of physiology and artificial intelligence is understanding how complex activity and behavior emerge from the intrinsic underlying structure and dynamics. To address this problem, we need new methodologies and tools to perform a comprehensive analysis of complex systems dynamics. Multifractal formalism and methodology enable us to investigate local interactions underlying physiological systems and quantify the organization of physiological temporal fluctuations and their cascades across scales (Plamen Ch. Ivanov et al., 1999, 2001; Ivanov et al., 2002; Mukli et al., 2015). Besides, we need a general network framework to examine networks of interactions among diverse subsystems across space and time scales that lead to emergent complex behaviors at the systems level (Bashan et al., 2012; P. C. H. Ivanov et al., 2016; Ivanov and Bartsch, 2014). Despite recent progress in the theory of dynamic networks, there are fundamental methodological and conceptual challenges in understanding how global states and functions emerge in networks of diverse dynamical systems with time-varying interactions and the basic principles of their hierarchical integration. In particular, when mining the time-varying complex networks structure and dynamics, one has to overcome various internal or external perturbations that can transiently or permanently mask the activity of particular nodes and their causal interactions (Gupta et al., 2019; Gupta et al., 2018; Xue and Bogdan, 2017a; Xue and Bogdan, 2019; Xue and Bogdan, 2017b).

Novel artificial intelligence techniques and machine learning algorithms may equip us with the tools to classify and predict the emergent behavior in dynamical networks based simultaneously on network topology and temporal patterns in network dynamics. Key insights and knowledge that emerge from multifractal and differential geometry concepts can help analyze and quantify their complexity. Furthermore, they allow us to determine the most efficient network architecture to generate a given function, quantify key universalities, and identify new theoretical directions for artificial intelligence and machine learning based on physiological principles (Richards et al., 2019). Ultimately, we will attain sustainable systems that enjoy seamlessly indistinguishable features of physiological systems (Wu et al., 2021).

From genomic, proteomic, and metabolic networks to microbial communities, neural systems, and human network physiology of organ systems, complex systems display multi-scale spatiotemporal patterns that are frequently classified as non-linear, non-Gaussian, scale-invariant, and multifractal (Bassingthwaighte et al., 2013; Ivanov et al., 2009; Stanley et al., 1999; West and Zweifel, 1992). While several efforts have demonstrated that electromyographic signals possess fractal properties (Sanders et al., 1996; Xue et al., 2016; Garcia-Retortillo et al., 2020; Rizzo et al., 2020), (Martin del Campo Vera and Jonckheere) report a complex bursting rate variability phenomenon where the surface electromyographic (sEMG) bursts are synchronous with wavelet packets in the D8 sub-band of the Daubechies 3 (db3) wavelet decomposition of the raw signal. Their db3 wavelet decomposition analysis reconstructs the sEMG bursts with two high coefficients at level 8, indicating a high incidence of two consecutive neuronal discharges. In contrast to heart rate variability (P. Ch Ivanov et al., 1998), the newly reported bursting rate variability phenomenon involves a time-localization of the burst with a statistical waveform matching between the “D8 doublet” and the burst in the raw sEMG signal. While this analysis focused on an available small cohort of patients, further comprehensive studies can elucidate the interdependencies between the electromyographic signals and other brain and physiological processes, determine the mechanistic role, and implications for medical applications.

Quests for understanding the inner workings of complex biological dynamics have provided not only more appropriate and efficacious medical therapies but have also led to new artificial intelligence algorithms and architectures. For instance, inspired by early modeling of how biological neurons process information, the reservoir computer model–a type of recurrent neural network where the set of outputs are fit to a training signal–provided promising high-performance low-power consumption computational strategies in classification tasks. Along these lines, (Carroll) considers the computational difficulty of parameter optimization in a reservoir computer and demonstrates that the optimum classification performance occurs for the hyperparameters that maximize the entropy of either a spiking reservoir computer or a reservoir computer. Intriguingly, T. Carroll shows that optimizing for entropy only requires a single realization of each signal to be classified, which provides a fast and low power computational strategy.

Intelligence is an essential trait characteristic of healthy biological systems, allowing them not only to locally optimize in search for better fitness states, but also to cope with unknown rare environmental perturbations. While much of the complex dynamical systems theory focused on defining, quantifying, and analyzing the degree of emergence (Balaban et al., 2018; Koorehdavoudi and Bogdan, 2016), self-organization (Balaban et al., 2018; Koorehdavoudi and Bogdan, 2016; Polani, 2008), self-optimization (Gershenson et al., 2021; Koorehdavoudi and Bogdan, 2016; Prokopenko et al., 2009; Prokopenko et al., 2014) and complexity (Adami, 2002; Jost, 2004; Koorehdavoudi and Bogdan, 2016) of various complex biological systems towards providing a definition of “intelligence” (Hernández-Orallo et al., 2021), generalization–the ability of a system to handle unexpected (future) situations for which it was not trained with a similar degree of success to that small data on which it was trained—remains an essential feature distinguishing human and artificial intelligence. Current efforts in artificial intelligence and machine learning investigate the degree to which a variety of neural network architectures are capable of “generalizing” and exhibiting intelligent behavior. Along these lines, Stoop) provides a series of fundamental examples demonstrating that for situations not included in the training efforts, the AI systems tend to run into substantial problems. These fundamental examples highlight not only the difference between human and artificial intelligence but also call for renewed interest in defining the theoretical foundations of intelligence.

By taking inspiration from biological neural systems capable of solving complex multi-objective problems characterized by ill-conditioned Hessians, Chatterjee et al. pioneer a fractional time series analysis framework that can not only model the neuro-physiological processes but also can circumvent the challenges of current optimization tools. More precisely, they show that the long-range memory observed in many biological systems and neurophysiological signals in particular exhibits non-exponential power-law decay of trajectories that can model the behavior associated with the objective function’s local curvature at a given time point. This allows them to propose the NEuro-inspired Optimization (NEO) method to deal with ill-conditioned Hessian problems. While promising, this effort shows that mathematical approaches targeting understanding the multifractality of biological systems can provide new theoretical directions for artificial intelligence.

The works presented in this Research Topic collection and current advances in the field of fractal and multifractal investigations of physiological systems structure and dynamics, and their applications to artificial intelligence, outline new challenges and opportunities in multidisciplinary research and applications. Dealing with the heterogeneity, multi-modality, and complexity of physiological and artificial systems requires rigorous mathematical and algorithmic techniques to extract causal interdependencies between systems across different scales while overcoming various noise sources. As such, progress in this direction will require new algorithmic strategies to quantify time-varying information flow among diverse physiological and artificial processes across scales and determine how it influences the system dynamics.

Furthermore, there is an urgent need to adopt a cross-scale perspective and a corresponding theoretical framework to investigate the multi-scale regulatory mechanisms underlying the overall network and its relation to emergent states and functions in physiological and artificial systems. This urges the interactions of statistical physics, non-linear dynamics, information theory, probability and stochastic processes, artificial intelligence, machine learning, control theory and optimization, basic physiology, and medicine, such that new theoretical and algorithmic foundations will emerge for analyzing and designing physiological and artificial systems. Only then, the biomedical and engineering communities will be able to develop new control methodologies that do not seek to only enforce a specific reference value but rather ensure that the complexity and multifractality are restored to a desirable profile.
